# Heel prick test: maternal-fetal conditions that may have an effect on the test results in newborns admitted to the intensive care unit

**DOI:** 10.5935/0103-507X.20190030

**Published:** 2019

**Authors:** Letícia Pinto Rodrigues, Sarah Cristina Sato Vaz Tanaka, Vanderlei José Haas, Valéria Cardoso Alves Cunali, Alessandra Bernadete Trovó de Marqui

**Affiliations:** 1 Universidade Federal do Triângulo Mineiro - Uberaba (MG), Brasil.; 2 Programa de Pós-Graduação em Atenção à Saúde, Universidade Federal do Triângulo Mineiro - Uberaba (MG), Brasil.; 3 Disciplina de Pediatria, Universidade Federal do Triângulo Mineiro - Uberaba (MG), Brasil.; 4 Disciplina de Genética, Universidade Federal do Triângulo Mineiro - Uberaba (MG), Brasil.

**Keywords:** Neonatal screening, Infant, newborn, Infant, premature, Mother and child health, Metabolism, inborn errors, Intensive care, neonatal, Intensive care units, Triagem neonatal, Recém-nascido, Recém-nascido prematuro, Saúde materno-infantil, Erros inatos do metabolismo, Terapia intensiva neonatal, Unidades de terapia intensiva

## Abstract

**Objective:**

To describe the characteristics of the heel prick test in newborns admitted to the intensive care unit of a university hospital as well as to determine whether maternal and fetal conditions could have affected the results of this test.

**Methods:**

Retrospective longitudinal study with a quantitative approach that evaluated 240 medical records. The data collected were analyzed by descriptive statistical analysis.

**Results:**

There was a predominance of pregnant women aged 20 to 34 years who had a complete secondary education and who had more than six prenatal care visits. Maternal complications or pathologies occurred in 60% of the mothers, and most (67.5%) did not present any condition that could have affected the heel prick test results. Most newborns were premature and exhibited low birth weight. Approximately 90% of newborns exhibited conditions that could have influenced the test, especially prematurity, parenteral nutrition and blood transfusion. Of the 240 newborns, 25% had abnormal heel prick test results, especially for cystic fibrosis and congenital adrenal hyperplasia.

**Conclusion:**

There are maternal and neonatal conditions that can affect heel prick test results, and therefore, their investigation is essential, aiming to guide measures that promote mother and child health and consolidate neonatal screening in this population.

## Abstract (pt)

**Objetivo:**

Descrever as características do teste do pezinho dos neonatos atendidos na unidade de terapia intensiva de um hospital universitário, bem como verificar se existiam condições maternas e fetais que pudessem interferir no resultado desse exame.

**Métodos:**

Estudo retrospectivo longitudinal de abordagem quantitativa que avaliou 240 prontuários médicos. Os dados coletados foram submetidos à análise estatística descritiva.

**Resultados:**

Houve predomínio de gestantes com idades entre 20 a 34 anos, com Ensino Médio completo e que realizaram mais de seis consultas pré-natais. As intercorrências ou patologias maternas ocorreram em 60% das mães, e a maioria (67,5%) não apresentou nenhuma condição que pudesse interferir no resultado do teste do pezinho. A maioria dos neonatos era prematura e exibiu baixo peso ao nascimento. Cerca de 90% dos neonatos exibiram condições que poderiam influenciar no exame, principalmente prematuridade, nutrição parenteral e transfusão sanguínea. Dos 240 neonatos, 25% apresentaram resultado alterado no teste do pezinho, sobretudo para fibrose cística e hiperplasia adrenal congênita.

**Conclusão:**

Existem condições maternas e neonatais que podem interferir no teste do pezinho e, nesse sentido, sua investigação é imprescindível, visando direcionar ações que promovam a saúde materno-infantil e consolidem a triagem neonatal nessa população.

## INTRODUCTION

The heel prick test (HPT) is part of the National Neonatal Screening Program (*Programa Nacional de Triagem Neonatal* - PNTN) and has a preventive character; the main objective of the test is to identify metabolic disorders that may be asymptomatic in the first days of an infant's life.^(1)^

The diseases screened by the HPT can be treated successfully, but some diseases, when not diagnosed and treated early, can lead to intellectual disability or even death. Thus, early diagnosis directly affects the prognosis and improvement of the quality of life of affected individuals. In the state of Minas Gerais, HPT is performed by the Diagnostic Support Action and Research Center (*Núcleo de Ações e Pesquisa em Apoio Diagnóstico* - NUPAD) and screens for six diseases: phenylketonuria (PKU), congenital hypothyroidism (CH), hemoglobinopathies (Hb), cystic fibrosis (CF), congenital adrenal hyperplasia (CAH) and biotinidase deficiency (BTD).^(1,2)^ NUPAD recommends that for newborns retained in the maternity ward, the HPT should be performed on the third to the fifth days of life. Subsequent samples, i.e., second, third and fourth samples, should be collected at 10, 30 and 180 days, respectively. However, the need for collection of these samples is dependent on the clinical conditions (stable or unstable), weight (≥ 1,500g or < 1,500g) or gestational age (≥ 32 weeks or <32 weeks) of the newborn.

Despite its extreme importance for neonatal health, the lack of information from parents, relatives and health professionals due to lack of awareness of the importance of the test compromises the effectiveness of the HPT.^(3,4)^

According to the biological neonatal screening manual of the Ministry of Health, there are some situations that may compromise the interpretation of HPT results. Maternal conditions include hypothyroidism, steroid use (prednisone, dexamethasone and betamethasone), CAH or PKU, acute fatty liver of pregnancy, vitamin B_12_ deficiency, parenteral nutrition and red blood cell transfusions. Newborn conditions that may affect the test results include immaturity of the hypothalamic-pituitary-adrenal axis, hepatic enzyme immaturity, hypothyroidism, hypoxia, preterm birth, total parenteral nutrition, red blood cell transfusion, and use of dopamine and steroids.^(5)^

The current literature lacks information on the specifications of the HPT, especially in newborns admitted to the intensive care unit (ICU). This population, most often composed of premature infants, exhibits unstable clinical conditions that can affect HPT results. Thus, studies that establish the HPT profile in newborns in this context are relevant because they help in understanding its specificities. Moreover, considering that the health of a mother can influence the need for admission of a newborn to a neonatal ICU, the data obtained may contribute to the development of actions and strategies that help improve the quality of care provided to this population, aiming to promote mother and child health.

This study aimed to describe the characteristics of the HPT for newborns admitted to the ICU of a university hospital as well as to determine whether maternal and fetal conditions could have affected the test results.

## METHODS

This study was approved by the Research Ethics Committee of the *Universidade Federal do Triângulo Mineiro* (UFTM) under opinion number: 2,448,011 and CAAE number: 77798017.5.0000.5154.

This is a retrospective longitudinal study with a quantitative approach performed at the neonatal ICU of the *Hospital das Clínicas* of UFTM (HC-UFTM), Uberaba, Minas Gerais (MG), Brazil. At the time of the study, HC-UFTM served 27 municipalities that composed the *Triângulo Sul* macro-region of the state of MG. It was the only hospital that offered high-complexity carefully through the Unified Health System (Sistema Único de Saúde - SUS) and had 302 beds, 20 of which were for the neonatal ICU.

The data for the mothers and the newborns were collected by analyzing the medical records of newborns admitted to the ICU of HC-UFTM between January 2015 and December 2016. The HPT results were obtained from internal ICU records. The inclusion criteria were (1) admission to the neonatal ICU during the stipulated period; (2) application of the HPT during the hospital stay; and (3) complete data available in the medical records. Newborns who did not meet the criteria previously described were excluded from the study.

To collect data from the 240 medical records available at the HC/UFTM Medical Archive Service, a form was prepared by the researchers and validated by experts in the field. Exemption from informed consent was requested, as this is a retrospective study, with data obtained from secondary sources, and most newborns were not in clinical follow-up at the HC at the time of the study.

The analyzed variables were maternal age, number of prenatal care visits, education level, complications or pathologies, maternal conditions that could affect the HPT results, gestational age, birth weight, newborn conditions that could affect the HPT, and the HPT results. The obtained data were entered and validated in an Excel^®^ spreadsheet using a double-entry system. A descriptive statistical analysis was performed using *Statistical Package for the Social Sciences* (SPSS), version 20.0.

## RESULTS

The present study evaluated 240 medical records with the aim of characterizing the newborns admitted to the ICU of HC-UFTM in 2015-2016. Considering that the well-being of a newborn is closely related to maternal conditions, information related to the pregnancy and the health of the mother were obtained. The maternal and newborn characteristics are provided in tables 1 and 2, respectively. The average birth weight of the newborns was 1,991.6g.

Regarding maternal conditions that could have affected the HPT results, the majority (67.5%) did not present any situation that could interfere with the test results. Among the interfering conditions mentioned in the manual from the Ministry of Health, the use of steroids and hypothyroidism was found in 16.9% and 11.8% of cases, respectively. Moreover, smoking (37.6%), alcohol consumption (27.8%) and drug use (5.9%) were also conditions observed that could have affected the HPT results by predisposing the mother and newborn to preterm delivery.

**Table 1:** Maternal profile and conditions

Variables	n (%)
Age (years)	
≤ 15	4 (1.7)
16-19	35 (14.6)
20-34	175 (72.9)
≥ 35	26 (10.8)
Prenatal care visits	
None	8 (3.3)
1-3	41 (17.1)
4-5	42 (17.5)
≥ 6	149 (62.1)
Education level	
Incomplete elementary education	15 (6.2)
Complete elementary education	22 (9.2)
Incomplete secondary education	78 (32.5)
Complete secondary education	125 (52.1)
Presence of complications or pathology during pregnancy	
Yes	148 (61.7)
No	92 (38.3)
Complications or pathologies*	
Other†	81 (54.7)
High blood pressure	63 (42.6)
Urinary tract infection	31 (20.9)
Infectious diseases	15 (10.1)
Gestational diabetes mellitus	11 (7.4)
Diabetes mellitus	9 (6.1)

* Report of more than one condition; therefore, the total exceeds 100%;

† preeclampsia, hypothyroidism and obesity.

**Table 2:** Neonatal profile and conditions

Variables	n (%)
Gestational age (weeks)	
< 37	190 (79.2)
≥ 37	50 (20.8)
Weight (g)	
< 2.500	168 (70.0)
≥ 2500	72 (30.0)
Presence of neonatal conditions that could affect the HPT results	
Yes	223 (92.9)
No	17 (7.1)
Conditions*	
Prematurity	190 (85.2)
Parenteral nutrition	127 (56.9)
Red blood cell transfusion	108 (48.4)
Use of dopamine	19 (8.5)
Hypoxia	17 (7.6)

HPT - heel prick test.

* Newborns with more than one condition.

Of the 240 newborns analyzed, 25% (n = 60) showed abnormal results for diseases screened by the HPT (Table 3). There were no abnormal results for Hb.

**Table 3:** Heel prick test results

Pathologies	Abnormal results	Preterm	Term
Congenital hypothyroidism	3	3	0
Phenylketonuria	2	1	1
Cystic fibrosis	44	30	14
Biotinidase deficiency	2	1	1
Congenital adrenal hyperplasia	6	5	1
Cystic fibrosis + congenital adrenal hyperplasia	3	1	2
Total	60	41	19

Regarding newborn conditions that could have affected the HPT results, of the three newborns with abnormal results for CH, all were premature, and two used parenteral nutrition. Of the two newborns who had abnormal results for PKU, one was premature and underwent blood transfusion. Among the 44 infants with abnormal results for CF (73.3%), 14 were full term, of which eight required parenteral nutrition, seven required blood transfusion, two exhibited hypoxia, and two received dopamine. Some newborns had more than one condition described above. Of the 30 preterm newborns, 17 were using parenteral nutrition, 19 required blood transfusion, four received dopamine, and three had hypoxia. Of the two newborns that had abnormal results for BTD, one was premature. Of the six newborns with abnormal results for CAH, five were premature, of which one had a red blood cell transfusion, one received dopamine, and one used parenteral nutrition; the term newborn also used parenteral nutrition. Three newborns exhibited abnormal results for both CH and CAH, two of which received transfusions (one preterm and one term).

## DISCUSSION

The aim of the present study was to describe the HPT results in newborns admitted to the IC, as well as to investigate maternal and neonatal conditions that could have affected the test results. There are no published studies on this topic. However, there are consolidated data for newborn hearing screening (NHS).^(6-11)^ Two of these studies analyzed the NHS results for newborns who remained in the ICU,^(6,10)^ two related NHS results to prematurity^(8,11)^ and others described NHS results and the profile of mothers and newborns in a maternity ward^(9)^ or outlined the sociodemographic profile of mothers of newborns who underwent NHS in a university hospital.^(7)^

The present study identified a predominant maternal age group from 20 to 34 years, considered the ideal reproductive period. Our results were in agreement with those found in the literature.^(12,13)^ A study conducted in the state of Paraná showed that 54% of the mothers of premature newborns were in this age group and that 34% of the sample was younger than 19 years old, i.e., adolescents.^(14)^ In our study, only 16.3% of mothers were younger than 19 years old, a percentage similar to that reported in the literature.^(7,13)^ In our study, the extreme age groups (≤ 19 years and ≥ 35 years) were not very prevalent.

Regarding prenatal care, our results indicated that approximately 60% of the mothers fulfilled the number of prenatal visits recommended by the Ministry of Health, i.e., six or more. A study of mothers of newborns admitted to a public neonatal unit showed that 59.6% had six or more prenatal visits,^(15)^ which agrees with the data presented here.

Approximately 50% of the mothers had complete secondary education, which is higher than that reported in Rio Grande do Norte^(16)^ and the Federal District.^(15)^ A study conducted in a university hospital in Rio Grande do Sul showed that 27.79% had incomplete elementary education; 17.71% complete elementary education; 15.04% incomplete secondary education and 32.19% complete secondary education.^(7)^ Thus, the mothers in our sample had a higher education level.

Our study showed that approximately 60% of the mothers reported complications or pathologies during pregnancy, with a predominance of preeclampsia, hypothyroidism, obesity, high blood pressure and urinary tract infection. Two recent studies conducted in an ICU reported frequencies of complications during pregnancy similar to those found in the present study.^(15,17)^ In the first study, with preterm newborns, pregnancy complications occurred in 67.9% of the cases, and urinary tract infections were the most common, observed in 28% of pregnant women, followed by preeclampsia (17%). Among these, 9% had high blood pressure and 4% diabetes mellitus.^(17)^ The other study showed that most mothers (68.5%) exhibited pregnancy complications, with preeclampsia the most prevalent (26.3%) followed by urinary tract infection (22.8%).^(15)^ In another study, complications during pregnancy were reported by only 27.5% of the mothers, with urinary tract infection being the most common (78%); high blood pressure (2.9%) and diabetes (1.1%) were less frequent.^(9)^ Preeclampsia and urinary tract infection were also the complications most commonly observed by Santos et al.^(12)^

There was a predominance of preterm newborns (79.2%), a result similar to those observed in other studies (78.4%,^(18)^ 78.87%^(10)^ and 79.3%^(15)^ with newborns in the ICU. Births occurring before 37 weeks of gestation are considered premature or preterm births. A recent study^(19)^ showed that prematurity was the main cause of childhood death in Brazil. In 2015, childhood deaths due to endocrine, metabolic, blood and immune diseases ranked 14^th^, which is higher than its rank in 1990 (34^th)^.^(19)^ The predominance of premature newborns in our study is an expected result considering the data collection site.

The mean birth weight of newborns was lower than that reported in the literature (2,299g).^(10)^ The World Health Organization (WHO) defines low birth weight as newborns with a birth weight less than 2,500g, regardless of gestational age. According to the literature, the percentage of newborns admitted to the ICU with low birth weight ranged from 68.1% to 77.6%.^(15,18)^

In regard to neonatal conditions that may have affected the HPT results, prematurity, the use of parenteral nutrition and blood transfusions were the most observed, similarly to that reported in the literature.^(20,21)^

Among the maternal conditions that could have affected the HPT results, there was a significant number of mothers who used tobacco or alcohol during pregnancy. These rates were much higher than those reported in the literature.^(9,16)^ One of these studies showed that only 1.4% and 10.5% of the mothers used alcohol and tobacco during pregnancy, respectively.^(9)^ The use of these substances was approximately 10% in another study.^(16)^ Such conditions may have an indirect effect on the HPT results because they predispose mothers to premature birth. A recent literature review showed that the consumption of such substances may predispose newborns to low birth weight/height and prematurity.^(22)^ The use of illicit drugs during pregnancy had an average rate of 6% in a study that determined the profile of mothers and the outcomes of births,^(16)^ a finding that agrees with the results reported here. In our study, the use of steroids was also found during pregnancy, which may generate false-negative results for CAH and HC, due to the suppression of thyroid-stimulating hormone (TSH), T4 (thyroxine) and 17-hydroxyprogesterone (17-OHP).^(23)^

In the present study, preterm infants had a higher number of abnormal HPT results. This is in accordance with a previous study, which showed that the preterm newborn population had more abnormal results in NHS than did term newborns.^(8)^

Blood collection for the HPT in newborns admitted to the ICU is not a simple process. The immaturity of these newborns and the necessary therapeutic interventions can affect both sample collection and the interpretation of the results. Thus, the screening protocol for these individuals should minimize false-positive and false-negative results and, at the same time, provide reliable results with the lowest possible number of samples collected.^(23)^

In the present study, all newborns with abnormal HPT results for CH were preterm. Neonatal screening, in these cases, is challenging due to the immaturity of the hypothalamic-pituitary-thyroid axis that preterm infants exhibit. This physiological immaturity alters the TSH concentration and compromises the test results. None of the newborns with abnormal results for CH used dopamine; dopamine suppresses TSH production, which can lead to false-negative results. Because of these particularities, additional blood collections are necessary for CH screening in this population. Our study did not find abnormal HPT results for CH in term newborns, but it is important to note that conducting the test before the first day of life may result in the detection of a physiological increase in TSH, producing a false-positive result. Thus, it is important that the HPT be conducted within the recommended period to reduce complications in the interpretation of the results.^(21,24)^

The HPT for PKU should consider that the newborn has ingested enough milk so that phenylalanine is detected in the bloodstream. Thus, blood collection should be performed at least 48 hours after beginning breastfeeding, minimizing the possibility of abnormal results.^(25)^ The use of total parenteral nutrition can promote the elevation of multiple amino acids and compromise the HPT results for PKU.^(23)^ Although this condition is frequent in newborns admitted to the ICU, PKU was not observed in our study. Prematurity also seems to affect the HPT results for this condition and was observed in one of the newborns with an abnormal result for this disease.^(20)^

Regarding the diseases screened for using the HPT, our results showed that CF had the highest number of abnormal results. In Brazil, neonatal screening for CF is performed by quantifying the levels of immunoreactive trypsinogen (IRT) at two moments, with the second being conducted until 30 days of life.^(26)^ Low birth weight, hypoxia and premature delivery are associated with increased IRT levels, which may contribute to the high rate of false-positive HPT results for CF.^(23,27)^ It is noteworthy that most newborns in the present study had low birth weight and that most newborns with abnormal results for CF were premature. These factors, in addition to hypoxia, possibly influenced the high rate of abnormal results for CF in our series.

The literature reports that newborns admitted to neonatal ICUs subjected to blood transfusion may have abnormal HPT results for BTD and, if possible, blood collection for the HPT must be done before the start of transfusion, even if this occurs before the standard time recommended.^(28)^ None of the two cases with abnormal results for BTD in our study had a blood transfusion.

The HPT for CAH evaluates 17-OHP levels and is extremely sensitive to the newborn's gestational age. Therefore, preterm newborns should have their 17-OHP levels adjusted based on weight, in an attempt to minimize false-positive results.^(21)^ The use of parenteral nutrition, low birth weight and blood collection for the HPT before the recommended time also contribute to false-positive results.^(20)^ In our study, factors such as prematurity and use of parenteral nutrition were observed in newborns with abnormal results for this pathology.

It is important to note that all newborns with abnormal HPT results are referred for specific tests, which confirm or exclude the diagnostic hypothesis.

Despite being a well-established procedure in many countries, there is a lack of data available in the literature regarding the HPT in newborns admitted to the ICU, which made it difficult to discuss our results. However, this highlights the importance of this study and the need for additional studies to better understand this topic.

## CONCLUSION

There are maternal and neonatal conditions that can affect heel prick test results and, in this sense, their investigation is essential to guide measures for promoting mother and child health and consolidating neonatal screening in this population.
